# Physical activity enjoyment, exercise motivation, and physical activity in patients with heart failure: A mediation analysis

**DOI:** 10.1177/02692155221103696

**Published:** 2022-06-09

**Authors:** Leonie Klompstra, Pallav Deka, Luis Almenar, Dola Pathak, Elena Muñoz-Gómez, Raquel López-Vilella, Elena Marques-Sule

**Affiliations:** 1 Department of Health, Medicine and Caring Sciences, 4566 Linkoping University, Linkoping, Östergötland, Sweden; 2 College of Nursing, 3078 Michigan State University, East Lansing, MI, USA; 3 Heart Failure and Transplantation Unit, Department of Cardiology, Hospital Universitario y Politécnico La Fe, Valencia, Spain; 4 Centro de Investigación Biomédica en Red de Enfermedades Cardiovasculares (CIBERCV), Instituto de Salud Carlos III, Madrid, Spain; 5 Department of Statistics and Probability, 3078 Michigan State University, East Lansing, MI, USA; 6 Department of Physiotherapy, Physiotherapy in Motion, Multispeciality Research Group (PTinMOTION), 16781 University of Valencia, Valencia, Comunitat Valenciana, Spain

**Keywords:** Physical activity, motivation, enjoyment, heart failure, exercise

## Abstract

**Objective:**

To determine whether physical activity enjoyment mediated the association between motivation and physical activity in patients with heart failure.

**Design and setting:**

A cross-sectional study at the cardiology clinic in the university hospital in Valencia, Spain

**Subjects:**

A total of 134 patients with heart failure.

**Main measurements:**

Physical activity was assessed with the International Physical Activity Questionnaire, motivation was assessed with the Exercise Motivation Index and Physical Activity Enjoyment was assessed with the Physical Activity Enjoyment Scale.

**Analysis:**

Mediation analysis using Hayes’ PROCESS macro (Model 4) for SPSS.

**Results:**

The mean age of the sample was 70 ± 14 years, 47 patients were female (35%), and 87 patients were in New York Heart Association I/II (67%). A positive relationship was found between exercise motivation and physical activity (*t* = 4.57, *p* < .01) and physical activity enjoyment (*t* = 11.52, *p* < .01). Physical activity enjoyment was found to positively affect physical activity (t = 3.50, *p* < .01). After controlling for physical activity enjoyment, the effect of exercise motivation on physical activity changed from a significant to non-significant (*t* = 1.33, *p* = .89), indicating that enjoyment completely mediated the relationship between motivation and physical activity. Overall, 25% of the variation in physical activity was explained by the mediation model.

**Conclusions:**

Physical activity enjoyment mediates the relationship between exercise motivation and physical activity in patients with heart failure. This means that even highly motivated heart failure patients may not be physically active if they do not enjoy the physical activity.

## Introduction

Sedentary behavior is associated with an increased risk of negative health outcomes including cardiovascular disease and all-cause mortality.^1^ Lower level of physical activity including sedentary lifestyle behavior is common in the Western world and a cause of public health concern.^2^ The Physical Activity Guidelines emphasize the importance of reducing sedentary time, in addition to participating in regular moderate to high-intensity physical activity and exercise.^3^

Exercise training is a well-established non-pharmacological therapy for patients with heart failure.^4,5^ Despite being safe and improving outcomes, only a small proportion of patients with heart failure participate in cardiac rehabilitation or adhere to physical activity advice.^6^ Prominent factors associated with the sustainability of physical activity include self-motivation, physical activity enjoyment, and self-efficacy.^7^ In patients with heart failure, fully powered randomized controlled trials that provided access to exercise facilities and trainers to improve self-efficacy failed to achieve desired levels of sustained physical activity.^8,9^ It is, therefore, worth investigating the influence of other factors such as motivation and enjoyment on physical activity in this population. Patients with heart failure state enjoyment as an important consideration for engaging in physical activity and non-adherence to physical activity recommendations.^10^ However, our understanding of the influence of physical activity enjoyment on motivation and physical activity is limited and has not been previously investigated. Therefore, the present study aims to determine whether physical activity enjoyment mediated the association between motivation and physical activity in patients with heart failure.

## Method

This is a cross-sectional study.

Patients were considered eligible if diagnosed with heart failure (regardless of their ejection fraction), free from major psychiatric or neurocognitive conditions, 18 years of age or older, able to understand Spanish, and/or not have any cognitive impairment that would make it difficult to comprehend and fill out the questionnaire. All patients with heart failure that visited the cardiology clinic in a university hospital in Valencia, Spain, between July and November 2020 were invited to participate. Patients provided oral and written informed consent before filling out the questionnaires and help was provided when needed. The present study complies with the Declaration of Helsinki and was approved by the Regional Ethics Committee (2020-440-1).

*Physical activity* was assessed with the International Physical Activity Questionnaire short form (s-IPAQ). This form contains seven items for identifying the frequency and duration of light, moderate, and vigorous physical activity as well as inactivity during the past week. The frequency of activity is measured in days and duration in hours and minutes. The answers to the questions were transformed into metabolic equivalent of task (MET-minutes). The total physical activity score is the sum of vigorous, moderate, and walking physical activity scores. Typical s-IPAQ correlations with an accelerometer were 0.80 for reliability.^11^

*Exercise motivation* was assessed with the exercise motivation index. The index consists of 15 statements followed by a 5-point rating scale for each statement, ranging from 0 (not important) to 4 (extremely important). The index consists of three sub-scales: physical motivation, psychological motivation, and social motivation. The total score is the mean of the scoring on items on the scale. The index is reliable and valid.^12^

*Physical activity enjoyment* was assessed by the Physical Activity Enjoyment Scale. The scale consists of 16 items scored on a 5-point rating scale ranging from 1 (totally disagree) to 5 (totally agree). The total score could range between 16 and 80 points. The higher the total score relates to a higher level of enjoyment. The scale is reliable and valid.^13,14^

All continuous variables were found to be normally distributed using Kolmogorov–Smirnov tests. Descriptive characteristics of the participants have been presented as means  ±  standard deviation or as percentages.

Partial correlation coefficients were estimated to examine the associations among exercise motivation, physical activity, and physical activity enjoyment. Since the data was not normally distributed in the study, Spearman's correlation coefficient (*r*) was used to conduct a correlation analysis of study variables.

Hayes’ PROCESS macro (Model 4)^15^ for SPSS was used to analyze the mediating effect of physical activity enjoyment on the association between exercise motivation and physical activity. Using the Hayes method, the direct effect of the independent variable (motivation) on the dependent variable (physical activity) and the indirect effect through physical activity enjoyment on physical activity were analyzed. The total effect is the sum of the direct and indirect effects. The direct effect of covariates such as age, sex, time after diagnoses, comorbidities, education, marital status, or employment status was analyzed and if found significant, were included in the mediation model. Statistical analyses were performed using SPSS version 26.0, and the level of statistical significance was set at α = 0.05.

## Results

In total, 134 patients with heart failure participated in this study. Demographic, clinical, and physical activity characteristics are presented in Table 1.

**Table 1.** Demographic, clinical, and physical activity characteristics of 134 patients with heart failure.

	*N* = 134
Age (years), mean ± SD	70 ± 14
Female gender, n (%)	47 (35%)
Time since diagnose (months) median (Q1–Q3)	47 (10–84)
Education, n (%)	
Primary school, n (%)	74 (55%)
Secondary school, n (%)	32 (24%)
University, n (%)	28 (21%)
Marital status, n (%)	
In a relationship	99 (74%)
Employment status, n (%)	
Employed	21 (16%)
Unemployed	22 (16%)
Retired	91 (68%)
Comorbidities, n (%)	
Diabetes	39 (29%)
Hypertension	32 (24%)
Renal dysfunction	30 (22%)
COPD	12 (9%)
NYHA class	
I	16 (12%)
II	71 (55%)
III	29 (23%)
IV	13 (10%)
Physical activity intensity levels, n (%)^a^	
High physical activity (>3000 MET-minutes)	28 (21%)
Moderate physical activity (600–3000 MET-minutes)	39 (29%)
Low physical activity (<600 MET-minutes)	67 (50%)
Sedentary time in minutes a week (mean ± SD)^a^	576 ± 201
Exercise motivation^b^	2.32 ± 1.21
Physical motivation	2.70 ± 1.17
Psychological motivation	2.47 ± 1.44
Social motivation	1.75 ± 1.30
Physical activity enjoyment^c^	56 ± 15

COPD: chronic obstructive pulmonary disease; MET, metabolic equivalent of task; NYHA, New York Heart Association; Q1–Q3, quartile 1–quartile 3; SD, standard deviation.

^a^ Measured with the International Physical Activity Questionnaire short form. Frequency of activity is measured in days, and duration in hours and minutes transformed into metabolic equivalent of task (MET-minutes). Sedentary time is measured in minutes sitting a week.

^b^ Measured with the exercise motivation index. The index consists of 15 statements followed by a 5-point rating scale for each statement, ranging from 0 (not important) to 4 (extremely important). The total score is the mean of the scoring on items on the scale.

^c^ Measured with the Physical Activity Enjoyment Scale. The total score could range between 16 and 80 points.

Approximately one-fifth of patients reported high physical activity (>3000 MET-minutes) (at least 1 h/day of moderate-intensity physical activity or half an hour of vigorous-intensity physical activity), approximately one-third of patients reported moderate physical activity (at least 30 min of moderate-intensity physical activity on most days), and half of the patients reported low physical activity. On average, patients were sitting for 9 h and 36 min a day. Patients identified physical and psychological motivation as the most important and rated social motivation as the least important for exercise (Table 1).

A correlation analysis showed that motivation was significantly and positively correlated to physical activity and physical activity enjoyment. Additionally, physical activity enjoyment was significantly and positively correlated to physical activity. In the direct effect, age was significantly associated with physical activity and therefore taken as a covariate in the mediation analyses (Table 2).

**Table 2.** Correlation analyses between exercise motivation, physical activity enjoyment, and physical activity in patients with heart failure.

	Physical activity	Motivation	Enjoyment
Physical activity^a^	—	0.530**	0.659**
Motivation^b^	0.530**	—	0.718**
Enjoyment^c^	0.659**	0.718**	—

*p < .05; **p < .01.

^a^ Measured with the International Physical Activity Questionnaire short form. Frequency of activity is measured in days, and duration in hours and minutes transformed into the metabolic equivalent of task (MET-minutes).

^b^ Measured with the exercise motivation index. The index consists of 15 statements followed by a 5-point rating scale for each statement, ranging from 0 (not important) to 4 (extremely important). The total score is the mean of the scoring on items on the scale.

^c^ Measured with the Physical Activity Enjoyment Scale. The total score could range between 16 and 80 points.

**Figure 1.**
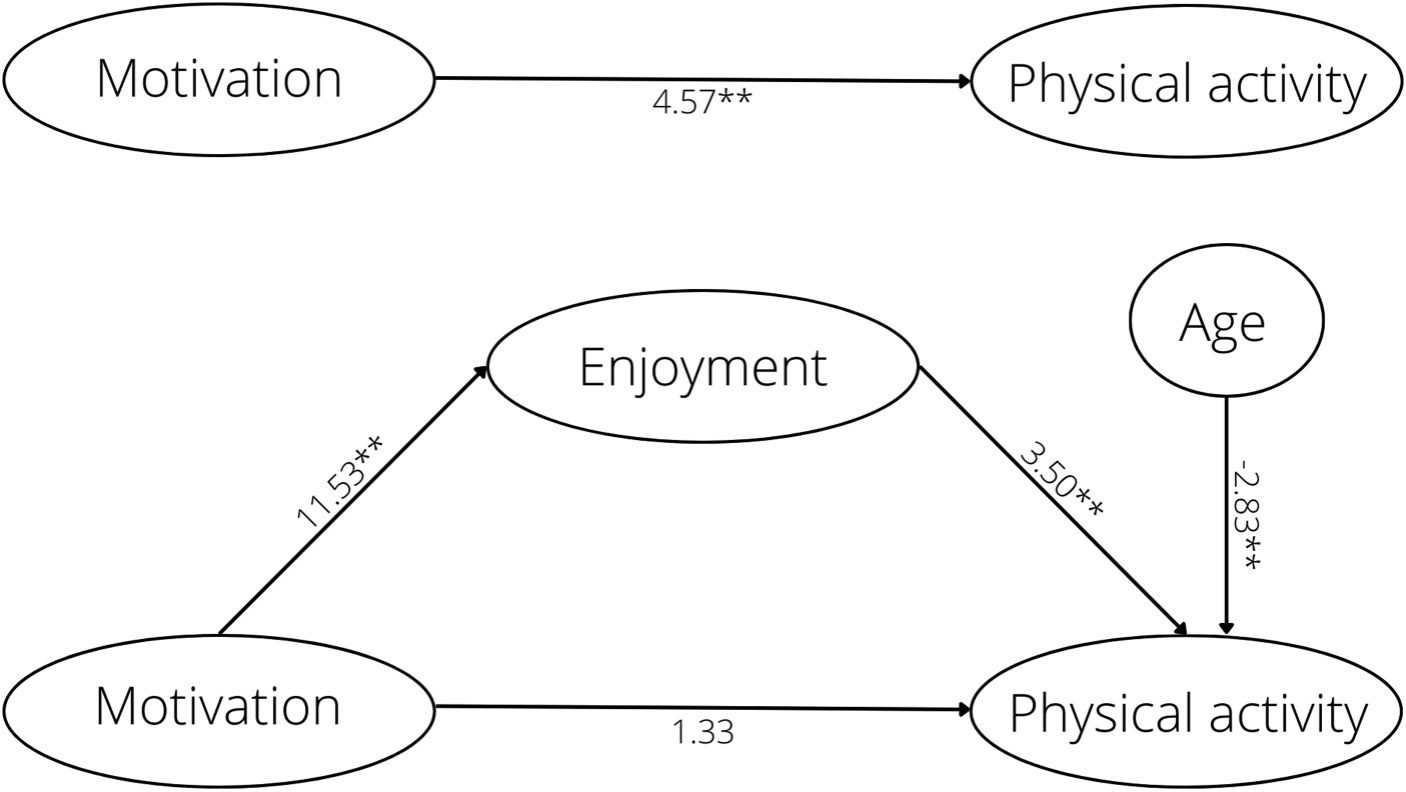
Analysis diagram of mediation effect of physical activity enjoyment on the relationship between exercise motivation and physical activity. Asterisks indicate significant coefficients (*p < .05; **p < .01).

Exercise motivation was positively associated with physical activity and physical activity enjoyment while physical activity enjoyment had a positive association with physical activity. After controlling for physical activity enjoyment, the effect of exercise motivation on physical activity changed from a significant to non-significant, indicating that enjoyment completely mediated the relationship between motivation and physical activity (Figure 1). Overall, 25% of the variation (*R*^2^) in physical activity was explained by the mediation model (*R* = 0.50, *F* = 13.09).

Physical activity was measured with the International Physical Activity Questionnaire short form. Frequency of activity is measured in days, and duration in hours and minutes transformed into the metabolic equivalent of task (MET-minutes).

Motivation was measured with the exercise motivation index. The index consists of 15 statements followed by a 5-point rating scale for each statement, ranging from 0 (not important) to 4 (extremely important). The total score is the mean of the scoring on items on the scale.

Enjoyment was measured with the Physical Activity Enjoyment Scale. The total score could range between 16 and 80 points.

## Discussion

To our knowledge, this is the first study that assessed the mediating role of enjoyment in the relationship between motivation and physical activity in patients with heart failure. In our sample, only 29% of patients (67% were in New York Heart Association-class I or II) reported being physically active and meeting the physical activity guidelines (30 min on most days in the week).^16^ We found that physical activity enjoyment completely mediated the relation between exercise motivation and physical activity. Our study highlights the fact that in becoming more physically active, patients need to be not only motivated but also recommended to engage in physical activity that they enjoy performing. Furthermore, we found that older age was associated with lower levels of physical activity.

Not enjoying physical activity should be considered an important barrier to being physically active in patients with heart failure. This is confirmed by interviews, where patients expressed that not being physically active was influenced by experiencing the physical activity as boring or not fun to perform.^10^ Other studies on cardiac patients found that enjoyment was not only important to becoming more physically active, but also important to sustaining long-term adherence.^17^ Similar findings have been reported on healthy adults where enjoyment during exercise mediated the effects of an exercise intervention on exercise adherence or the intention of continuing sports.^18,19^ A systematic review and meta-ethnography^20^ found that enjoyment can even play a more important factor in the elderly population as an awareness of mortality associated with aging may heighten the personal value placed on feelings of enjoyment.

Our results show that physical activity declines with age. This result is consistent with other empirical studies on the general population. Research suggests that the types of physical activities that older adults engage in differ markedly from the activities performed by younger adults.^21^ Findings from a qualitative study^10^ suggest that patients with heart failure may find enjoyment in engaging in the same activities as they did at a younger age but may need help and support in adapting its intensity to cope with their disease severity. Both previous and current physical activities should be considered in exercise advice and health care professionals could help patients to adapt their physical activity by using tools and resources or to find new ways of maintaining an active lifestyle. Examples of intensity-adapted activities for older adults can include walking football,^22^ modified dancing,^23^ or Medi-Yoga.^24^ Technology can also be utilized to help patients perform activities in their own home, like playing golf or tennis with help of an exergame^25^ or using video-conferencing to engage patients with heart failure with peers to improve exercise adherence.^26^

Our mediation model estimated 25% (*R*^2^) of the variation present in physical activity in patients with heart failure. The lower *R*^2^ can be explained by the fact that physical activity in patients with heart failure is influenced by many other factors such as self-efficacy, motivation, fear, anxiety, and depression.^7,27,28^ Other barriers to physical activity encountered by older adults include unaffordable physical activity programs, travel to an exercise facility, conflict with scheduling, or limited availability.^7^ Exercise facilities may not always have qualified professionals to supervise patients for their medical condition-specific exercise needs, and patients often report feeling like an outsider in an environment with younger attendees.^29^ Older patients with heart failure may also not enjoy the activities that young healthy adults do. The World Health Organization recently recommended strongly that safe and appropriate facilities are needed to promote physical activity for the elderly with chronic diseases.^30^ However, existing facility-based physical activity programs have not been able to meet the demand in this population.^29^ Therefore, community-based physical activity programs that patients are both motivated and enjoyed, should be developed, incorporating flexibility in the choice of various modalities of physical activities, and the possibility of support from peers and/or exercise coaches.^6,9,31^

A limitation of this study is the relatively small number of patients. Also, our sample consists of patients with heart failure living in Spain with a larger proportion of males than females. As such, any generalization should be made cautiously. Another limitation was that physical activity was self-reported, which is limited by recall bias and overestimation.

As long-term adherence to the recommended physical activity and exercise guidelines has been difficult to achieve, assessing enjoyment of physical activity seems to be of great importance. To increase physical activity in patients with heart failure, it is not only important to motivate patients, but also to give them the possibility to choose an exercise that they will enjoy. This observation is supported by the Self-Determination Theory, which postulates that improving the autonomy of choice for exercise can build intrinsic motivation for physical activity engagement and adherence.^32^

As the amount of physical activity is lower in the elderly patient with heart failure compared to younger patients, it is important to motivate these groups differently and to offer different physical activity programs to different age groups. Future studies should look at the effects of increasing enjoyment on physical activity and are advised to measure physical activity objectively.^33^

**Clinical messages**

- It is not only important to motivate patients with heart failure in becoming more physically active, but healthcare professionals should also investigate the types of physical activity that heart failure patients enjoy performing prior to recommending any exercise.
